# Synchronous Rotation Dynamics in a Molecular Motor

**DOI:** 10.1021/jacs.6c02815

**Published:** 2026-05-21

**Authors:** Robert Kluifhooft, Janna Wilhelmsen, Marco Kapitzke, Ann-Kathrin Rückert, Sergey A. Kovalenko, Ilya N. Ioffe, Julia Stähler, Michael Kathan, Samuel Palato

**Affiliations:** † Department of Chemistry, 9373 Humboldt-Universität zu Berlin, Berlin 12489, Germany; ‡ Department of Chemistry, Lomonosov Moscow State University, Moscow 119991, Russia; § Department of Physical Chemistry, 28259 Fritz-Haber-Institut der Max-Planck-Gesellschaft, Berlin 14195, Germany

## Abstract

Direct measurements of molecular motion during chemical reactions are essential to understand how molecular machines perform work. In most systems, however, the reaction rate is dictated by the probability of reaching the transition geometry by thermal fluctuations, thereby masking the underlying molecular motions. Here, we study the dynamics of rotation around the central double bond of an artificial light-driven molecular motor by femtosecond transient absorption and fluorescence spectroscopy. We observe a first rotation step, assigned to a rotation of ∼28° which occurs synchronously across the motor ensemble and without an activation barrier, such that the measurements are representative of molecular dynamics. We can thus estimate the rotation speed and the relative importance of inertia, friction and strain, and propose a simplified nanomechanical model for the molecular motor. The results suggest a new framework to investigate work at the nanoscale and provide tools to analyze the mechanics of molecular machines, both synthetic or biological.

## Graphical abstract

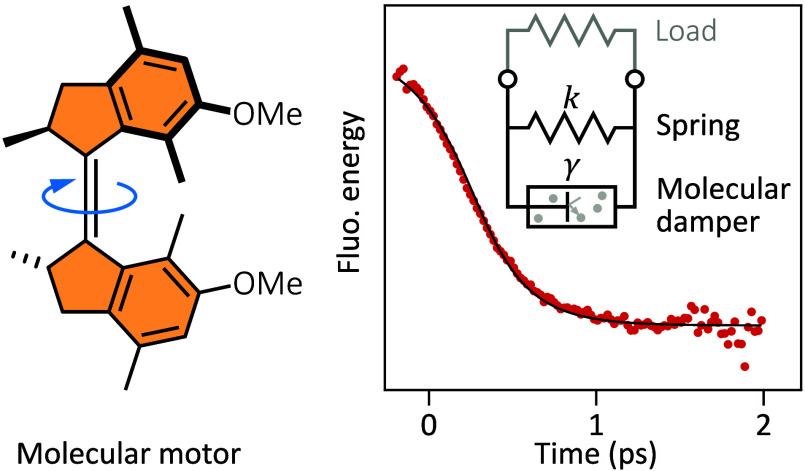

## Introduction

The study of molecular motion during chemical reactions is a long-standing topic of fundamental interest. Multiple approaches have been developed to understand these motions in the gas, solution, and solid phases: femtochemistry has encountered immense success in the gas phase,^1–3^ and ultrafast diffraction allows tracking of structural dynamics in solids.^4–8^ Investigations of reactions in solution have proven to be highly challenging due to the strongly dissipative environment.^9,10^ Applied to molecular motors (MMs),^11–18^ the experimental observation of molecular motion would yield insight into work at the nanoscale, and finely contrast microscopic and macroscopic behavior.^19–23^ Ideally, the behavior of molecular machines can be captured by simplified models, providing building blocks for composite designs and to understand the large biological engines powering life, such as kinesin and ATP synthase.^24^

The measurement of molecular motions during chemical reactions is made difficult for two reasons. First, the time scales involved are very fast, ranging from a few femtoseconds to picoseconds. This problem has been thoroughly solved by femtosecond spectroscopy techniques, at least when the reaction can be triggered by light. The second obstacle to the measurement of molecular motion arises from the statistics of large ensembles of molecules under study. For a measurement on the ensemble to be representative of the motion of individual molecules, the dynamics should occur synchronously across the ensemble. Figure 1A contrasts the probability distributions for synchronous and asynchronous processes. In a synchronous process, the probability distribution remains unimodal and the average evolves in a manner analogous to macroscopic mechanics. The motion of the average is representative of the motion of individual molecules. By contrast, most chemical reactions occur asynchronously: the rate of reaction is dictated by the probability of reaching the transition state and going “over-the-barrier”. As a result, the probability distribution is bimodal and follows chemical kinetics: the kinetics only indicate the successful crossings of the barrier, and all details regarding molecular motions are obscured. This problem is particularly acute in solution, a highly dissipative environment. Collisions with solvent molecules continuously randomize atomic velocities and dissipate excess energy, making synchronous motion all the more exceptional. Critically, the statistical behavior of the motion is a property of the sample and its potential energy surface: a barrierless trajectory can support trajectory-like motion, whereas a trajectory going over a high barrier produces chemical kinetics, regardless of how precisely the initial state is prepared. So far, experiments on MMs could not quantify molecular motion.

**Figure 1:**
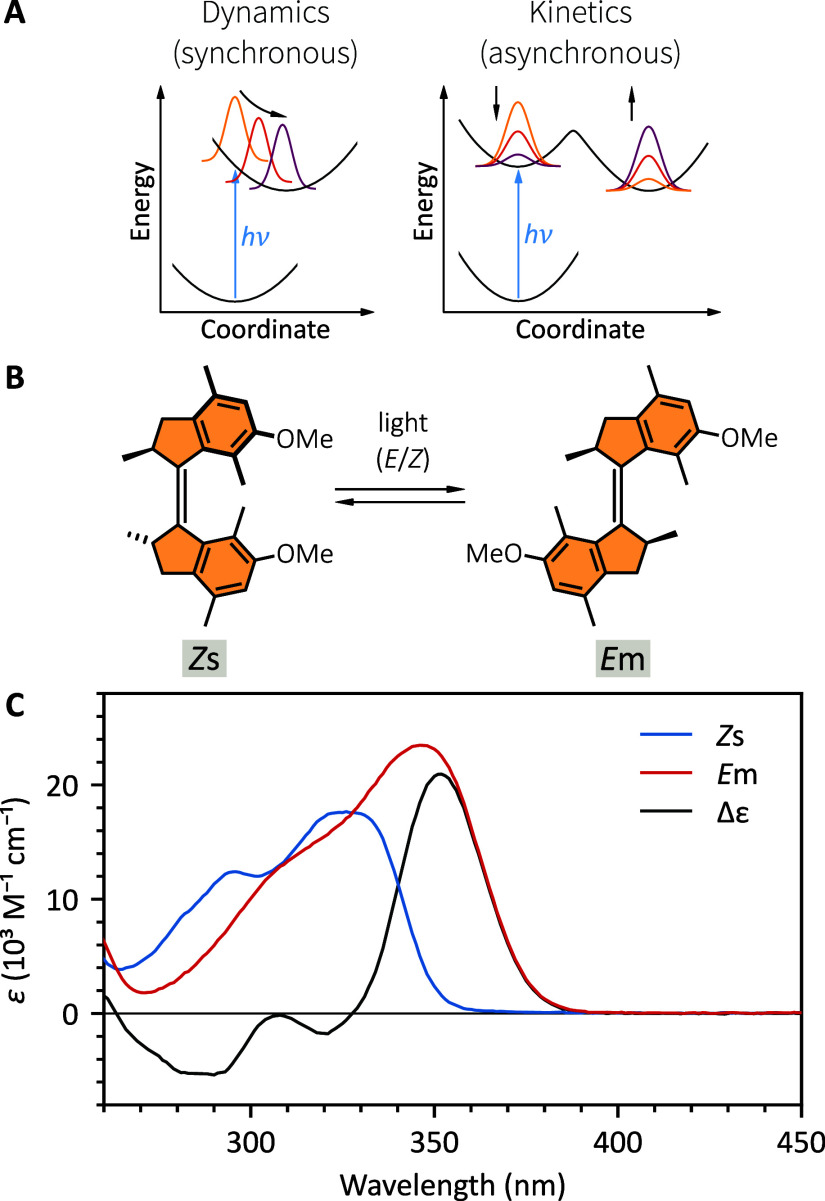
(A) Evolution of the probability distribution for synchronous dynamics compared to asynchronous chemical kinetics. (B) *Z*s to *E*m conversion of the molecular motor. Stiff-stilbene motif highlighted in color. Only the (*S*,*S*)-enantiomer is shown for clarity although the motor unit was used as a racemic mixture. (C) UV–vis spectra of the two species, *Z*s and *E*m in acetonitrile. The differential absorption spectrum (black) corresponds to the conversion of *Z*s to *E*m.

Here, we report on the observation and quantification of synchronous rotation dynamics during the first step of the *Z*/*E* isomerization of an artificial light-driven MM. Triggerable by light, this motor is investigated using femtosecond transient absorption (TA) and fluorescence upconversion spectroscopy (FLUPS). The results are interpreted through statistical mechanic modeling and detailed quantum mechanical calculations, enabled by its small size. The first step in the photoinduced reaction reveals a continuous peak shift occurring in the first 600 fs. The shift magnitude and dynamics are independent of solvent polarity; it is therefore due to intramolecular reorganization. Using quasi-degenerate multiconfiguration perturbation theory, we assign the continuous peak shift to the first step of rotation by about 28° around the central double bond which occurs without a barrier. The dynamics are modeled as an overdamped Brownian oscillator (OBO), and we therefore extract a microscopic friction coefficient for the rotation. The clear observation of continuous motion allows us to estimate the speed of the initial rotation step. We synthesize our results by suggesting a minimal nanomechanical model for the MM: a spring plus a molecular damper with negligible mass.

The MM studied here is an archetypal motif used in a variety of synthetic molecular machines. The molecule, shown in Figure 1B, is a first-generation Feringa-type MM, based on a stiff-stilbene core, which undergoes unidirectional rotation around the central double bond under UV illumination.^11,25^ The rotation cycle and synthesis of this motor has been previously described in detail elsewhere.^14,26^ This MM has recently been exploited in the design of molecular machines capable of synthesizing mechanically interlocked molecules such as catenanes and rotaxanes.^15,16^ This study focuses on the first photoinduced step, which converts the stable *Z* isomer (*Z*s) to the metastable *E* isomer (*E*m), shown in Figure 1B. The absorption spectra of the two isomers in acetonitrile are shown Figure 1C. The black curve shows the change in the absorption spectrum due to the conversion from *Z*s to *E*m. Monitoring the change in the absorption spectrum enables tracking of the reaction; femtosecond spectroscopy allows one to follow the isomerization process with femtosecond resolution.

A recent review summarizes the current understanding of Feringa-type unidirectional MMs.^27^ They follow a pattern shared with stilbenes: the nascent excited state relaxes within a few hundred fs to a dark state via torsion around the central double bond and pyramidalization of the ethylenic carbons.^28^ This dark, perpendicular-pyramidalized intermediate then decays on ps time scales to the ground-state reactant and photoproduct through an intersection. Throughout the process, the dihedral angle around the central double bond increases and serves as the reaction coordinate. *Z*/*E* isomerization in *Z*-stiff-stilbene is understood similarly, though two excited-state geometries were identified: first a partially twisted excited *Z* intermediate, then a perpendicular-pyramidalized intermediate.^29–31^ Some studies have reported an equilibrium between these two intermediates, though the results are understood to depend on the molecule and the environment.^31,32^ Beyond chemical kinetics, there have been a few reports of synchronous relaxation in these systems, where a continuous peak shift shift was observed and interpreted as excited state relaxation.^29,33,34^ However, these studies general lacked either time resolution or spectral bandwidth to fully resolve the dynamics.

The experimental findings are supported by quantum-chemical calculations encompassing highly accurate calculations of stationary points, mapping of the potential energy surface, and dynamical simulations.^30,35,36^ Stilbenes and the derived MMs are challenging to model due to the relatively flat PES, with several states of comparable energy, and exhibit considerable static and dynamic correlation. Computationally fast methods such as TDDFT generally fail to identify the local minima on the PES. Accurate calculations therefore require multireference quasi-degenerate perturbation theories, which are numerically expensive. This has stimulated the development of tailored approaches to map the PES with sufficient accuracy.^35–39^ The conclusions are generally compatible with experiment: the global minimum of the S_1_ PES lies at or close to the perpendicular configuration and is dark. Depending on the molecule and method, local minima may exist close to the *Z* and *E* configurations. PES mapping reveals that the central dihedral angle plays a determining role in the isomerization process, its evolution being remarkably linear throughout the entire cycle, while the two neighboring dihedral angles are strongly anticorrelated with it.^36^ In summary, experiments and QCC on Feringa-type MMs and stilbenes show that relaxation proceeds through one or a few excited states with increasing torsion around the central double bond.

## Results and Discussion

We investigate isomerization dynamics using TA and FLUPS, which track absorption and fluorescence after photoexcitation with fs resolution and thereby monitor excited-state processes.^27,29,31,33,40–44^ The methods are complementary: both probe the same dynamics but with different contrast. FLUPS detects only fluorescent (“bright”) states, while TA also captures “dark” states and ground-state depletion. Instantaneous fluorescence is directly related to the TA stimulated emission (SE) signal via the Einstein coefficients, so FLUPS appears as a negative TA contribution, and agreement between the two confirms the validity of the measurements. In both methods, peak shifts indicate excited-state energy changes, and peak-area changes track populations and, when applicable, oscillator strength changes. Experimental details are provided in the Supporting Information. Together, they reveal both continuous reorganization and chemical kinetics through multiple possible intermediates.

Figure 2 compares TA and FLUPS results after 325 nm excitation. The TA spectra (top row) contain several overlapping features. During the first 600 fs (A), a negative band G matches the inverted *Z*s absorption spectrum (light gray) and corresponds to depletion of the *Z*s ground state, or ground state bleach (GSB). Then, around 340 nm, a positive band P develops during the first 600 fs, similar to a known signal in stiff-stilbene. In the same range, there is also a negative contribution labeled SE. Negative TA signal can have two origins: ground state depletion and SE. As it does not overlap with the *Z*s absorption spectrum, this feature is assigned to SE. It shifts toward longer wavelengths during the first 600 fs. Finally, an excited state absorption (ESA) feature, spanning from ∼450 nm to the end of our detection window is observed. It shifts similarly to SE. The TA spectra of the first 600 fs are, therefore, dominated by the shift of the SE and ESA features, with concurrent growth of P.

**Figure 2:**
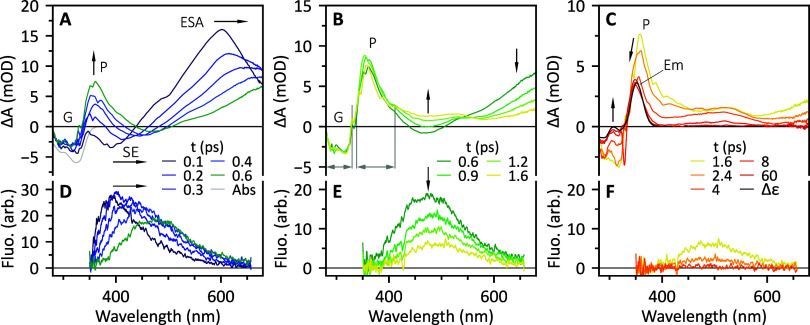
Femtosecond absorption and fluorescence track the isomerization process. (A–C) Differential absorption at selected delays after excitation. (D–F) Fluorescence spectra at the same delays. All results in acetonitrile. The light gray spectrum in (A) shows the absorption spectrum of *Z*s, flipped and scaled. The black curve in panel (C) shows the differential absorption spectrum corresponding to the photoisomerization.

Figure 2B shows the evolution of the TA spectra between 600 fs and 1.6 ps. The G and P features remain mostly unchanged. The SE feature decays and the signal becomes positive. Similarly, the ESA feature decays. Figure 2C shows the final evolution of the TA signal (*t* > 1.6 ps). The G feature decays incompletely, indicating partial ground state recovery. The P feature decays, shifts slightly and narrows. After 8 ps, the TA spectrum matches the *E*m-*Z*s difference spectrum (black line in Figures 2C and 1C), confirming the conversion of *Z*s to *E*m. Overall, the TA data indicate three time scales, matching the 3 panels: (A) subpicosecond peak shifts of SE and ESA and growth of an intermediate P; (B) ps-scale changes in excited state composition; and (C) final decay to ground state *Z*s and *E*m within 8 ps.

The multiple overlapping features in the TA spectra complicates the analysis. We therefore complement our measurements of the excited state dynamics with FLUPS. For ease of comparison, the fluorescence signals have been converted to their equivalent SE signal (*I*_SE_(λ)∝λ^4^ *I*_*F*_(λ)) in Figure 2.

The FLUPS spectra in Figure 2D show a single broad fluorescence band, shifting from a peak of 400 to 475 nm in the first 600 fs. This shift closely mirrors the shift of the SE band in TA. The spectrum decays homogeneously across intermediate and long time scales (Figure 2E,F). The decay of the FLUPS signal in panel E is consistent with the changes of the TA signal from negative to positive in the same spectral region. The fluorescence has completely disappeared after 4 ps, indicating a depletion of the fluorescing excited state.

The qualitative inspection of the TA and FLUPS spectra above already reveals the following: The G feature of the TA spectra closely matches the inverse of the ground state *Z*s spectrum, while the TA spectra at long delays match the difference spectrum between the *E*m to *Z*s, confirming the experiment probes the photoexcitation of *Z*s and the final production of *E*m. Both SE and FLUPS signals shift to longer wavelengths within 600 fs, corresponding to a stabilization of the excited state. The distinct time scales for the decay of the FLUPS signal and ground-state depletion signal from TA indicate that more than one excited intermediate is present: the slower dynamics are due to “dark” excited states.

We now analyze the fluorescence signal quantitatively. It is analyzed first as it is simpler and a subset of the observables. Figure 3A shows the band integral of the FLUPS signal over the entire bandwidth, proportional to the population and oscillator strength of the emitting state. The band integral follows a single exponential decay profile, indicating a single population of emitting molecules decaying with a time constant τ_1_ = 0.8303(20) ps. The evolution of the FLUPS signal therefore indicates a single population of excited molecules whose energy is shifting on femtosecond time scales.

**Figure 3:**
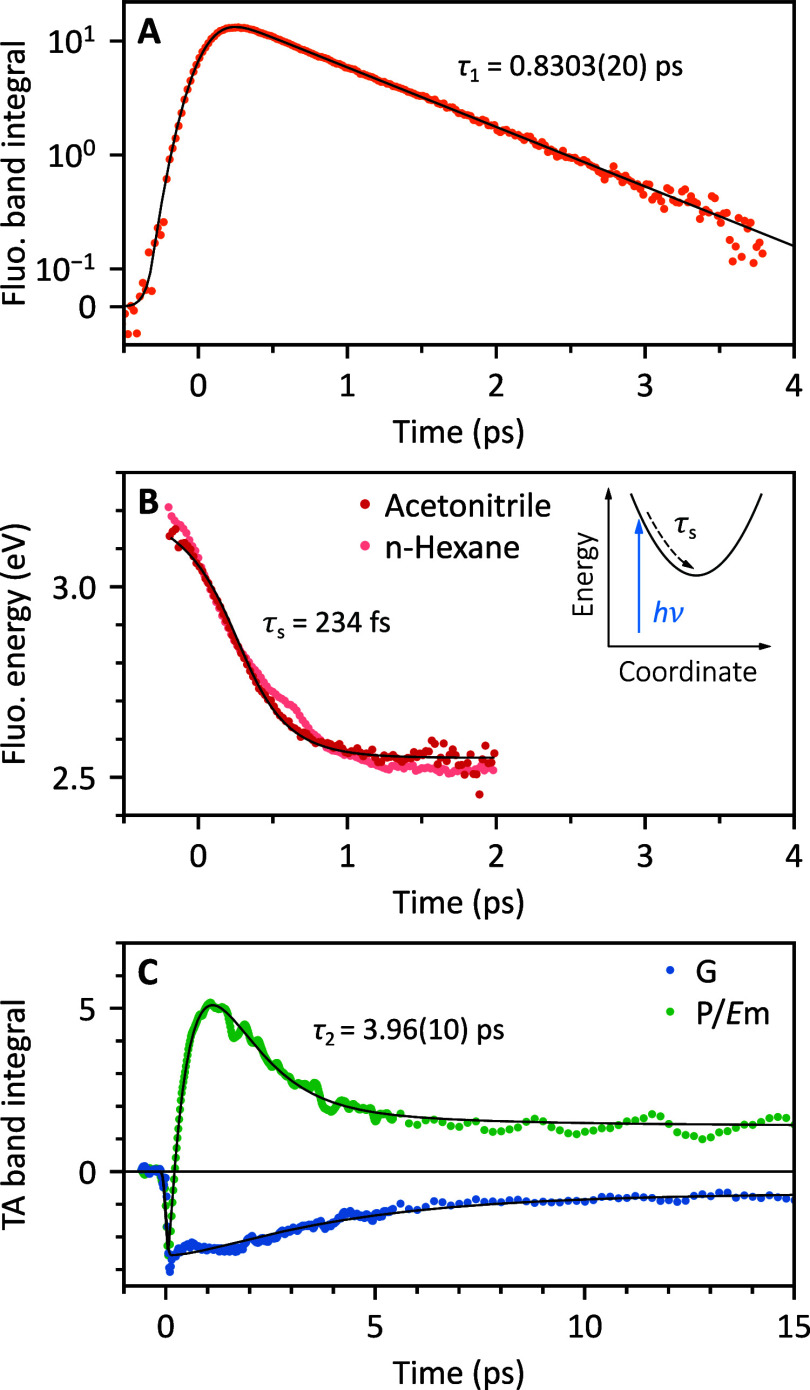
Analysis of the photoinduced dynamics. (A) Fluorescence decay shows a single decay component. Scale linear below 10^–1^. (B) The average fluorescence energy shifts with time constant 234 fs. The shift is the same in polar (acetonitrile) and nonpolar solvents (*n*-hexane), indicating it has an intramolecular origin. Inset: schematic representation of the overdamped Brownian oscillator model. (C) Global analysis of the population kinetics from TA, showing formation and decay of the intermediate P and return to the ground state.

Figure 3B shows the shift of the fluorescence energy. The fluorescence energy is measured as the barycenter of the emission line shape vs energy (see Supporting Information section S8). We use energy instead of wavelength in this case as it is more directly related to the properties of the molecule and to model calculations. The fluorescence shifts continuously by more than 0.6 eV within the 600 fs. Such continuous peak shifts are commonly observed in the case of solvation dynamics, where it is due to reorganization of the solvent to accommodate a change in the charge distribution of the molecule in the excited state. This process is very well understood: the magnitude of the shift increases with the polarity of the molecule and the polarity of the solvent.^45,46^ The dynamic shift is therefore small to nonexistent in nonpolar solvents, and increases in magnitude with solvent polarity. We compare the peak shift observed in acetonitrile (strongly polar) and *n*-hexane (nonpolar) in Figure 3B. Clearly, they are very similar, with an even slightly larger magnitude in the nonpolar solvent *n*-hexane. Therefore, we can rule out solvation as the origin of the peak shift. Instead, the dynamic shift of the fluorescence energy must correspond to intramolecular reorganization.

We model the behavior using an OBO model where the dynamics arise from a harmonic oscillator subject to the tandem forces of fluctuations (“thermal kicks”) and dissipation (friction), neglecting inertia.^47–50^ The inset to Figure 3B shows a scheme of this model; the black line in Figure 3B shows the model results for acetonitrile. The time-dependent FLUPS spectrum is modeled using the cumulant expansion approach, accounting for the experimental time resolution and population decay shown in Figure 3A (see Supporting Information section S7). It indicates a relaxation time constant τ_s_ = 0.234 ps and a total energy shift of 716 meV. The use of two modes would be underdetermined as the data shows a single relaxation time constant. Remarkably, a single OBO mode is sufficient to explain the peak shift.

Having analyzed shift and population dynamics within the first picoseconds using FLUPS, we now analyze the subsequent steps using TA. Figure 3C shows the kinetics of the band integrals corresponding to the G band and P/*E*m band. The exact limits are indicated by gray lines in Figure 2B. Both band integrals are fitted to the same kinetic model consisting of an initial excited state *Z*s, an intermediate state P and the final ground states *Z*s and *E*m (see Supporting Information section S9). Using τ_1_ from FLUPS, a global fit yields τ_2_ = 3.96(10) ps for the decay of the P state into *Z*s and *E*m. The population dynamics on ps time scales are therefore quantitively consistent with FLUPS and follow a kinetic scheme similar to stiff-stilbene and related MMs.^27,29,31–33,51–55^

We now summarize the experimental results. The first process is a relaxation of the nascent *Z*s* to a relaxed *Z*s* geometry with τ_s_ = 234 fs, indicated by the shift of the FLUPS and SE signals. The shift occurs with a single time constant, and is therefore described by a single effective mode. There is no corresponding decay of the FLUPS signal, therefore this motion does not reduce the oscillator strength. This result is in contrast with second generation fluorene-based MMs, where relaxation makes the state completely dark,^33,37^ but in line with results on stilbenes and stiff-stilbene-based molecular motors, where the oscillator strength is constant or partially reduced.^29,35,41^ The relaxed *Z*s* state then evolves into a P intermediate with τ_1_ = 0.8303 ps, as indicated by the decay of the FLUPS band integral. Our results in *n*-hexane shows two exponential components (see Supporting Information section S7), which suggests the presence of a *Z*s* ⇄ P equilibrium in nonpolar solvents. Such equilibria were previously reported in stilbenes, and the results consistent with partial charge transfer character of P.^31,34,32,35,29,30^ Finally, the P state decays back to the *Z*s and *E*m ground states with τ_2_ = 3.96 ps. As the population kinetics are well understood, they shall not be discussed further.

In order to further quantify the microscopic dynamics, we turn to quantum mechanical calculations. The relaxed geometry of the ground and excited state and the vertical excitation spectra were obtained by means of the XMCQDPT2 quasi-degenerate multiconfiguration perturbation theory as implemented in the Firefly package v. 8.2.1, which is partly based on the GAMESS­(US) software^56–59^ (see Supporting Information section S10). Figure 4A shows the ground state geometry. The dihedral angle around the central double bond (4 labeled atoms) is 3.0°. Puckering of the 5-membered rings enable the aromatic rings to twist out of plane and minimize steric repulsion. Figure 4B shows the relaxed excited state geometry, configuration B.^60^ As typical for stilbenes, the S_1_ state is based on the single-electron HOMO–LUMO excitation.^29,30,40^ The HOMO is bonding with respect to the central C=C bond and antibonding with respect to the adjacent C–Ph bonds while the LUMO has exactly the opposite structure. Accordingly, excitation to the S_1_ state results, in particular, in weakening and elongation of the central bond and allows an increase in the central dihedral angle to 30.7° and diminution in the adjacent ones between the C=C bond and the phenyl planes. Flexibility around the double bond allows the two aromatic rings to align and overlap, presumably to favor π stacking. Altogether, the initial excitation essentially preserves the molecular shape and volume. Neither geometry shows pyramidalization of the ethylenic carbons. The configuration B is located Δ*E* = 0.45 eV below the nascent excited state and has a vertical emission energy of 2.66 eV, in good agreement with our experimental results (2.55 eV). In summary, detailed quantum mechanical calculations reveal a relaxed excited state geometry associated with a rotation of ∼28° around the central double bond.

**Figure 4:**
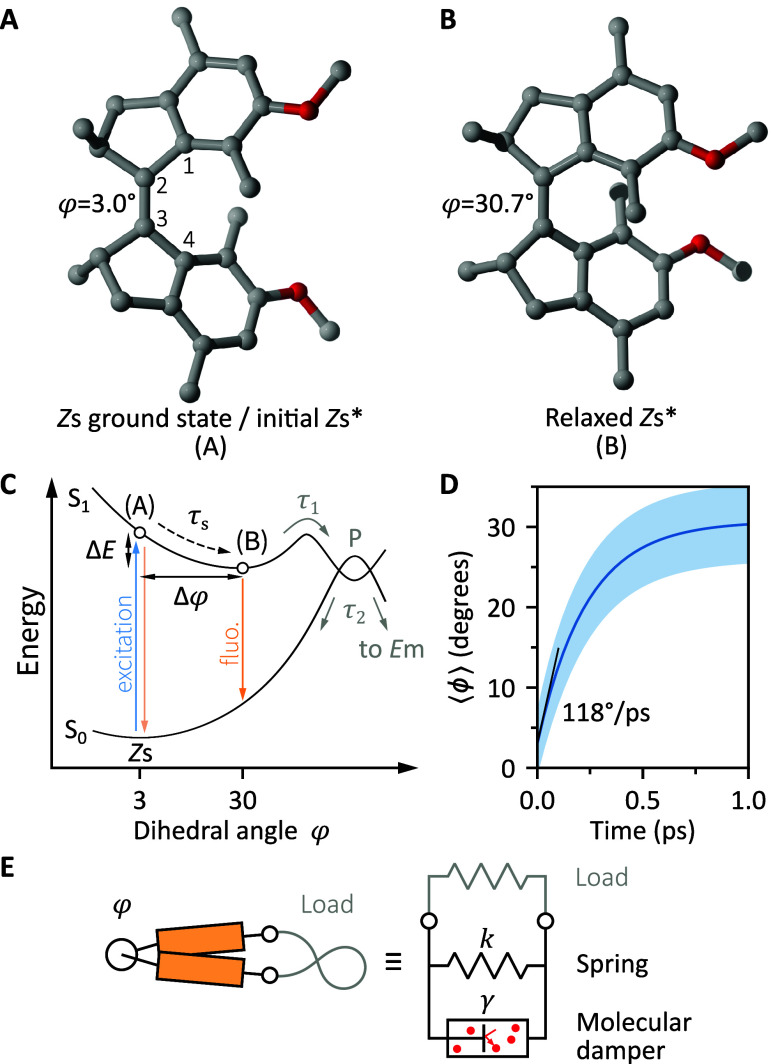
Microscopic rotation dynamics. (A) Ground state geometry. The dihedral angle formed by the labeled atoms is nearly planar. (B) Relaxed excited state geometry. The main distortion is the rotation by ∼28° around the double bond. (C) Dynamics illustrated on a potential energy surface. The peak shifts correspond to a relaxation between the ground state and the relaxed excited state geometries (A, B). (D) Trajectory of the average dihedral angle (line) and 1σ width (shaded area) of the overdamped Brownian oscillator. (E) Minimal nanomechanical model of the molecular motor: spring and molecular damper.

## Microscopic Dynamics

We clearly and quantitatively resolve the initial continuous intramolecular reorganization, which is obscured by asynchronous kinetics in other MMs. We can therefore estimate the speed of rotation and analyze the balance of conservative force, fluctuation and dissipation. Figure 4C illustrates the photoinduced dynamics on a PES. According to the Born–Oppenheimer approximation, the excited state molecules are initially in the ground-state geometry (A). They then relax toward geometry (B), a local minimum of the excited state PES. We thus assign the initial intramolecular stabilization to an average rotation of Δφ = 27.7° around the central double bond, which occurs without a barrier and with a time scale τ_s_ = 234 fs. From B, the molecules then evolve by standard thermally activated kinetics toward an intermediate state P, and subsequently to the ground state *E*m and *Z*s.

Figure 4D shows the trajectory of the average dihedral angle obtained using the OBO model. As inertia is negligible, the system does not have an acceleration phase, the bond rotates at up to 118°/ps on average immediately after excitation and slows down exponentially. Similarly, it dissipates energy at an average rate up to 1.92 eV/ps/molecule. The initial motion is caused by thermal fluctuations, not steric repulsion. Together with the compact intermediate geometry, this indicates that twisting of the MM is not launched by steric repulsion as it would be from a compressed spring^19^ but instead the potential biases the fluctuations, as demonstrated by the equation of motion below.

We can summarize the behavior of the MM using an equivalent mechanical model formed by a minimum number of basic elements, called a lumped-element or mass-spring-damper model in mechanical engineering. These models allow the derivation the systems equations of motion from a simplified description.^61,62^ Our model is a reduced description of the MM consisting of a single mode φ, and is represented in Figure 4E. The MM is equivalent to a simple spring in parallel with a molecular damper. The spring corresponds to the force due to the gradient of the PES and has a spring constant *k* = 2Δ*E*/Δφ^2^ = 1.17 meV/deg^2^ for the initial rotation. The molecular damper corresponds to the friction acting to damp the motion. In contrast to its macroscopic equivalent, the molecular damper is responsible for both damping and fluctuations. It dissipates energy in excess of *k*_*B*_ *T*, but does not stop the motion (indeed, it ensures constant agitation). The friction coefficient is γ = τ*k* = 0.274 meV ps/deg^2^, and the fluctuations of the force have a standard deviation of $\sqrt{2k_{B}T\gamma }$ . This minimal model has no mass: inertia is negligible compared to the other forces. This yields the following effective stochastic equation of motion for the motor (see Supporting Information section S11)^63,64^

$$\mathrm{\varphi ̇}=-\frac{k}{\gamma }\left(\varphi -\varphi_{0}\right)+\sqrt{\frac{2k_{B}T}{\gamma }}\eta \left(t\right)$$

where η­(*t*) is uncorrelated Gaussian noise with variance of 1. In physical terms, the first term drives the molecule toward the equilibrium geometry, while the second term represents the perpetual agitation by the thermal environment. The simplified model allows for a rigorous treatment of the statistical aspects. For example, the evolution of the width of the distribution is known (shaded area in Figure 4D, see Supporting Information section S11). In analogy with mechanical and electronic engineering, we hope that combining multiple lumped-elements will facilitate the study of large MMs and MMs under load, if the individual elements can be parametrized from QCC or experiments.^16,27^

The single-mode description is a simplified, reduced description of the system: the actual nuclear motion involves many coordinates simultaneously. However, a model with more modes would not be supported by the data. We use the dihedral angle as a dominant coordinate, as it is known to increase monotonically throughout the isomerization process. The parameters *k* and γ are therefore effective coefficients that capture the behavior of all coupled motions projected onto this coordinate. The coefficient γ reflects contributions from both the solvent and intramolecular degrees of freedom to fluctuations and dissipation. A systematic variation of solvent viscosity would be required to disentangle the intra- vs intermolecular contributions. The rotation around the central double bond is compensated by the counter-rotation of the neighboring dihedral angles. This correlated motion of a large number of atoms has a low probability, and is a likely source of intramolecular fluctuations and dissipation. Modern QCC methods capable of mapping the excited-state PES could quantify the degree of coupling between these coordinates, and test this interpretation. Despite the suspected complexity of the molecular motion, a simple model with only one mode fully describes the behavior. Based on this surprisingly simplifying insight, we suggest that even large and complex machines can be composed of such simplified models.

## Conclusion

In conclusion, we have observed continuous rotation around the central double bond in a light-driven molecular motor. The first step in the *Z*s to *E*m isomerization is a continuous barrierless rotation of ∼28° around the central double bond to a relaxed geometry (B) which is synchronized across the ensemble. The rotation is modeled as an overdamped Brownian oscillator, which leads us to propose a minimal mechanical model for the working of this motor, consisting of solely a spring and a molecular damper. The current measurements were performed on the motor only, in the so-called no-load conditions. However, the observability of the dynamics enables studies of the molecular motor under load and promises an unprecedented window of investigations into work at the molecular scale.

## Supplementary Material



## Data Availability

Supporting files and exact software versions used in this manuscript are available at 10.5281/zenodo.17912190. All other data are available in the main text or the Supporting Information.

## Notes

Detailed synthesis, characterization, and PSS determination; experimental details, data processing in FLUPS; details of fitting of FLUPS intensity, FLUPS peak shift, and TA population kinetics; details on quantum-chemical calculations; derivation of the effective equation of motion (PDF)
