# Supplementary material for: Synchronous Rotation Dynamics in a Molecular Motor
Source: J Am Chem Soc. 2026 May 21;148(21):21826–33. doi: 10.1021/jacs.6c02815 (PMC13244437; doi:10.1021/jacs.6c02815)
Supplement: Supplementary file 1 [file ja6c02815_si_001.pdf]

Supporting Information for: Synchronous rotation dynamics in a molecular motor.

Robert Kluifhooft[a], Janna Wilhelmsen[a], Marco Kapitzke[a], Ann-Kathrin Rückert[a], Sergey A. Kovalenko[a], Ilya N. Ioffe[b], Julia Stähler[a,c], Michael Kathan[a], Samuel Palato[a]*

[a] Department of Chemistry, Humboldt-Universität zu Berlin; Berlin, 12489, Germany

[b] Department of Chemistry, Lomonosov Moscow State University; Moscow, 119991, Russia

[c] Department of Physical Chemistry, Fritz-Haber-Institut der Max-Planck-Gesellschaft; Berlin, 14195, Germany

Corresponding author: samuel.palato@hu-berlin.de

Main article: https://doi.org/10.1021/jacs.6c02815

Contents

S1. Synthesis – Detailed Characterization 2

S2. PSS Determination and irradiation experiments 3

S3. Femtosecond spectroscopy, general remarks 4

S4. Transient absorption. 4

S5. Fluorescence upconversion 4

S6. Fluorescence upconversion: data processing 5

S7. Fitting of the fluorescence decay intensity 5

S8. Modeling of the fluorescence peak shift 6

S9. Population kinetics in TA 7

S10. Details of quantum-chemical calculations 9

S11. Derivation of the effective equation of motion 10

References 11

Supplementary figures 13

S1. Synthesis – Detailed Characterization

Reagents were purchased as reagent grade and used without further purification, unless otherwise specified. All non-aq. reactions were performed in oven-dried glassware and under Ar atmosphere. Automated Medium Pressure Column Chromatography (MPLC) was performed on a Teledyne ISCO CombiFlashRf 300 system with 200 mL/min max flow, 200 psi, equipped with integrated ELSD and 200–800 nm UV-vis variable wavelength detector. Thin layer chromatography (TLC) was conducted on aluminium sheets coated with SiO_2-60 F_{254} obtained from Merck; visualization with a UV lamp (254 or 365 nm). Nuclear magnetic resonance (NMR) spectra were recorded using a Bruker Avance II 500 (500 MHz for ^1H, 126 MHz for ^{13}C) at 25 °C and are reported as follows: chemical shift (δ) in ppm (multiplicity, coupling constant J in Hz, number of protons; assignment). The residual deuterated solvent was used as the internal reference. For ^1H NMR: $CDCl_3$ δ_H = 7.26 ppm and for ^{13}C NMR $CDCl_3$ δ_C = 77.16 ppm. The resonance multiplicity is described as s (singlet), d (doublet), t (triplet), q (quartet), p (pentet), m (multiplet), and br (broad). Ultra-performance liquid chromatography (UPLC) was performed on an Acquity H-class UPLC equipped with an Acquity QDa detector in combination with a PDA eλ diode array detector using reversed phase columns (Acquity UPLC BEH phenyl cyclohexyl (1.7 μm 100 × 2.10 mm) or Acquity UPLC BEH C18 (1.7 μm 50 × 2.10 mm)). High resolution mass spectrometry (HRMS) was performed on a Xevo G3 QTof MS, measuring in either positive or negative mode. Ultraviolet-Visible (UV-vis) absorbance spectroscopy was performed on Agilent Cary 60 instruments connected to a cryostat from Unisoku Scientific Instruments (temperature accuracy ± 0.1 K) in 10 × 10 mm quartz cuvettes with 3 mL volume. Weighing of small quantities was performed on a Sartorius ME5 analytical microbalance. The extinction coefficient of motor *Z*s at 325 nm was determined using a dilution series and was found to be $17.6 \cdot 10^3$ mol^{-1} cm^{-1} at 20 °C.

(*Z*)-6,6'-Dimethoxy-2,2',4,4',7,7'-hexamethyl-2,2',3,3'-tetrahydro-1,1'-biindenylidene and (*E*)-6,6'-Dimethoxy-2,2',4,4',7,7'-hexamethyl-2,2',3,3'-tetrahydro-1,1'-biindenylidene

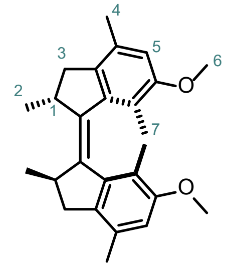

*Z*s
$C_{26}H_{32}O_2$
Mw =376.5311

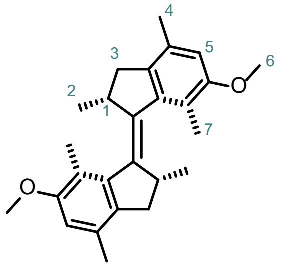

*E*s
$C_{26}H_{32}O_2$
Mw =376.5311

This synthesis was based on a literature procedure[1–3]. To a suspension of zinc powder (0.10 g, 1.5 mmol, 4.0 equiv.) in anhydrous 1,4-dioxane was added $TiCl_4$ (0.08 mL, 0.14 g, 0.74 mmol, 2.0 equiv.). The reaction mixture was heated at reflux for 2 h, after which a solution of 6-methoxy-2,4,7-trimethyl-2,3-dihydro-1H-inden-1-one (0.10 g, 0.37 mmol, 1.0 equiv.) in 1,4-dioxane was added and the resulting mixture was heated at 66 °C for 5 d. The reaction was

quenched by addition of aq. HCl (1 M, 20 mL) and the product was extracted with EtOAc. The combined organic layers were washed with a sat. aq. $NaHCO_3$, brine, dried over Na_2SO_4 and concentrated under reduced pressure. The crude product was purified by MPLC (SiO_2, cHex/EtOAc gradient 100:0 ⟶ 98:2) to give the *E*/*Z* motor as a yellow solid (41%). The *E*s motor isomer was obtained by recrystallization of the mixture from EtOH, while the *Z*s motor isomer was obtained after thermal isomerization of the mother liquor in nonane at 160 °C for 3 d with subsequent crystallization from EtOH.

(*Z*s): ^{1}**H NMR** (600 MHz, $CDCl_3$, 25 °C) δ = 6.53 (s, 2H, H^5), 3.78 (s, 6H, H^6), 3.33 (p, *J* = 6.7 Hz, 2H, H^1), 3.04 (dd, *J* = 14.5, 6.3 Hz, 2H, H^3), 2.38 (d, *J* = 14.5 Hz, 2H, H^3), 2.26 (s, 6H, H^4), 1.37 (s, 6H, H^7), 1.07 (d, *J* = 6.7 Hz, 6H, H^2) ppm.

^{13}**C{^{1}H} NMR** (151 MHz, $CDCl_3$, 25 °C) δ = 156.5, 142.3, 141.1, 136.1, 130.6, 122.1, 110.4, 55.9, 42.0, 38.2, 20.6, 19.0, 14.3 ppm.

HR-ESI-TOF-MS (ESI+) m/z calculated for $C_{26}H_{33}O_2^+$ ([M+H]$^+$), 377.2475 found 377.2477. Mass error: 0.5 ppm.

(*E*s): ^{1}**H NMR** (600 MHz, $CDCl_3$, 25 °C) δ = 6.56 (s, 2H, H^5), 3.86 (s, 6H, H^6), 2.89 (p, *J* = 6.3 Hz, 2H, H^1), 2.60 (dd, *J* = 14.1, 5.7 Hz, 2H, H^3), 2.30 (s, 6H, H^4), 2.20 (s, 6H, H^7), 2.16 (d, *J* = 14.1 Hz, 2H, H^3), 1.09 (d, *J* = 6.5 Hz, 6H, H^2) ppm.

^{13}**C{^{1}H} NMR** (151 MHz, $CDCl_3$, 25 °C) δ = 157.0, 142.6, 141.8, 134.1, 131.4, 120.4, 110.0, 55.9, 42.4, 38.5, 19.3, 18.9, 16.3 ppm.

HR-ESI-TOF-MS (ESI+) m/z calculated for $C_{26}H_{33}O_2^+$ ([M+H]$^+$), 377.2475 found 377.2477. Mass error: 0.5 ppm.

NMR spectra are shown on Fig. S2 to S5 at the end of this document.

Data is in accordance with that reported in literature[2].

S2. PSS Determination and irradiation experiments

The rotation of motor *Z*s was followed by UV/vis experiments. Fig. S6 shows the rotation cycle. Fig. S7 shows the spectra taken for PSS determination. Fig. S8 shows the spectra taken during the rotation cycle in MeCN. Fig. S9 shows the spectra taken during the rotation cycle in n-hexane. From these experiments the PSS ratios between the *Z*s and *E*m state and *E*s and *Z*m state were determined using UPLC-HRMS. Metastable states were always relaxed to the stable states before injection. Samples were eluted on an Acquity UPLC BEH Phenyl (2.1 × 100 mm) column using a MeCN/Water/IPA (9:2.5:1 + 0.1% formic acid) mixture as the mobile phase.

To obtain reliable integration with UPLC-HRMS, detection has to be performed at a wavelength where the extinction coefficients of both states are equal (i.e. at the isosbestic point). To determine the isosbestic point, a UV/vis spectrum of *Z*s (~20 μL in MeCN/Water/IPA 9:2.5:1 + 0.1% formic acid) was measured at 20 °C and subsequently irradiated using Thorlabs M310L1

(308 nm, 38.5 mW) mounted LED until PSS. The *Z*m state was then irradiated using Thorlabs M405L2 (405 nm, 410 mW) mounted LED to form the *E*s state. The UV/vis spectrum after irradiation was recorded at 20 °C. Overlapping the *Z*s and *E*s spectra as shown on Fig. S7 gives the isosbestic point of 326 nm in the UPLC mixture.

S3. Femtosecond spectroscopy, general remarks.

All experiments were performed on solutions of the compound in UVAsol-grade solvents. The solution was circulated using a home-built flow-cell with thin UVFS windows. Absorption spectra of the solution were taken before, during and after the measurements to control for composition evolution. The time scans were performed in alternating forward and backward direction. The data was inspected for composition evolution, which was found to be negligible for transient absorption. Evolution was more significant in FLUPS, leading us to keep only the first pair of scans.

S4. Transient absorption.

Our transient absorption spectrometer has been described in detail elsewhere[4]. The instrument uses a Ti:Sapphire amplifier (Coherent Astrella, 5 kHz, 35 fs, 800 nm). A portion of the CPA output drives a tunable optical parametric amplifier and harmonic generator (Light Conversion TOPAS-Prime and NIRUVis) set to an output wavelength of 325 nm to generate the pump beam. Another part of the CPA output is frequency-doubled to 400 nm and used for white-light generation in a translating CaF_2 plate. The pump beam polarization was set to magic angle. The instrument uses chopping and single-shot referencing to improve signal-to-noise ratio. The excitation energy was 128 nJ/p. The measurements were performed with timesteps of 20 and 200 fs and each delay scan repeated 8 times to improve signal to noise, with alternating scan directions. The sample was in a flow cell with thickness ~200 µm.

S5. Fluorescence upconversion.

The FLUPS spectrometer has been described in detail before[5,6]. The output of a Ti:Sapphire amplifier (Coherent Astrella, 5 kHz, 35 fs, 800 nm) drives two tunable optical parametric amplifiers and harmonic generators (Light Conversion TOPAS, Light Conversion TOPAS-Prime+NIRUVis) set to 325 nm for the excitation beam and 1340 nm for the gate. The excitation beam is focused on the sample flow cell (thickness: ~500 µm). The fluorescence and gate beam are focused onto a BBO crystal in case B geometry with a ~22° crossing angle. The upconverted signal is then detected by a spectrometer and high gain detector (Andor Newton EM). The delay of the gate with respect to the excitation beam is scanned in step of 20 fs using a retroreflector mounted on a mechanical delay line (Physik Instrumente). Multiple scans are performed in alternating directions to improve signal to noise. Wavelength is calibrated using a Hg lamp. The intensity response of the apparatus is calibrated using the dyes C6H, C153 and PBBO in acetonitrile as secondary standards. The reference dye solutions were measured in a calibrated fluorescence spectrometer. The dispersion correction curve was obtained by reference measurements on PBBO and BBOT in acetonitrile.

S6. Fluorescence upconversion: data processing

Due to evolution of the composition during the experiment, a photodynamical background due to the *E*s isomer is observed to increase with scan number. Its contribution is removed in two ways: first, only the initial two scans from a fresh solution are kept, leaving only a residual 5% contribution to the initial fluorescence intensity. This residue is then removed by DAS, using the known kinetics of *E*s measured by TA (no spectral dynamics, single exponential behavior, τ=11.9 ps).

For comparison with the TA spectrum, the FLUPS spectra are converted to their equivalent stimulated emission as $S_{\mathrm{SE}}(t,\lambda) = \lambda^4 S_{\mathrm{F}}(t,\lambda)$, therefore including both the ω^2 factor for spontaneous vs stimulated emission and the Jacobian for energy to wavelength conversion. The lineshape was computed as $f(t,E) = S_{\mathrm{SE}}(t,E)/E$, and the barycenter as:

$$\langle E(t)\rangle = \frac{\int E\, f(t,E)\mathrm{d}E}{\int f(t,E)\mathrm{d}E} \tag{1}$$

S7. Fitting of the fluorescence decay intensity

As previously mentioned, the time-resolved fluorescence spectra are converted to the equivalent stimulated emission as $S_{\mathrm{SE}}(\lambda,t) = \lambda^4 S_{\mathrm{F}}(\lambda,t)$. The band integral computed as:

$$I(t) = \frac{1}{\ln(\lambda_2/\lambda_1)} \int_{\lambda_1}^{\lambda_2} \frac{S_{\mathrm{SE}}(\lambda,t)}{\lambda} \mathrm{d}\lambda \tag{2}$$

is then proportional to the emitting population and oscillator strength. The band integral is fitted to the following model:

$$I_F(t) = F(t) * K(t) \tag{3}$$

$$F(t) = \frac{1}{\sigma_t\sqrt{2\pi}} \exp\left[-\frac{1}{2}\left(\frac{t-t_0}{\sigma_t}\right)^2\right] \tag{4}$$

$$K(t) = \sum_i a_i \exp\left(-\frac{t}{\tau_i}\right) \tag{5}$$

where $F(t)$ is a Gaussian IRF of width σ_t and position t_0, $K(t)$ is a multiexponential decay profile and $*$ indicates convolution along t. A single component is sufficient for acetonitrile, and two components are required for n-hexane. The parameters are shown in Table S1.

Table S1. Fit parameters for population decay in FLUPS. Numbers in parenthesis indicate the standard error on the last two digits (1σ).

Solvent	$\boldsymbol{\sigma_t}$ (ps)	$\boldsymbol{a_0}$	$\boldsymbol{\tau_0}$ (ps)	$\boldsymbol{a_1}$	$\boldsymbol{\tau_1}$ (ps)
acetonitrile	0.13878(65)	18.534(35)	0.8303(20)	-	-
n-hexane	0.1574(10)	12.04(45)	0.585(27)	11.30(55)	1.965(55)

S8. Modeling of the fluorescence peak shift

The peak shift of the fluorescence spectrum is modeled using the cumulant expansion to second order for a two-level system, following the work of Mukamel and Myers-Kelley[7–10]. This method enables the modeling of time-dependent spectra using transition dipole moments, transition energies and lineshape functions. The cumulant expansion models the impulsive response without relaxation. The effect of experimental time resolution and population decay are then included, thereby producing a generative model for our FLUPS measurement. We first describe the cumulant expansion modeling, then describe the inclusion of finite time resolution and population decay.

Using the cumulant expansion, the time dependent fluorescence is obtained from the third order response:

$$R_{\mathrm{F}}(t_1, t_2, t_3) = R_1(t_1, t_2, t_3) + R_2(t_1, t_2, t_3) \tag{6}$$

where:

$$R_1(t_1, t_2, t_3) = \left|\mu_{eg}\right|^4 \exp\left[-i\omega_{eg}(t_3 + t_1)\right] F_1(t_1, t_2, t_3) \tag{7}$$

$$R_2(t_1, t_2, t_3) = \left|\mu_{eg}\right|^4 \exp\left[-i\omega_{eg}(t_3 - t_1)\right] F_2(t_1, t_2, t_3) \tag{8}$$

and:

$$\begin{aligned} F_1(t_1, t_2, t_3) = \exp[&-g^\star(t_3) - g(t_1) - g^\star(t_2) + g^\star(t_2 + t_3) + g(t_1 + t_2) \\ &- g(t_1 + t_2 + t_3)] \end{aligned} \tag{9}$$

$$\begin{aligned} F_2(t_1, t_2, t_3) = \exp[&-g^\star(t_3) - g^\star(t_1) + g(t_2) - g(t_2 + t_3) - g^\star(t_1 + t_2) \\ &+ g^\star(t_1 + t_2 + t_3)] \end{aligned} \tag{10}$$

where $g(t)$ is the lineshape function. In general, it is related to the spectrum of energy fluctuations $C(\omega)$ via:

$$g(t) = -\frac{1}{2\pi}\int_{-\infty}^{\infty} \frac{C(\omega)}{\omega^2}\left[e^{-\mathrm{i}\omega t} + \mathrm{i}\omega t - 1\right] \tag{11}$$

For the overdamped harmonic oscillator, the lineshape function is known:

$$g(t) = g_{\mathrm{R}}(t) + \mathrm{i}g_{\mathrm{I}}(t) \tag{12}$$

$$g_R(t) = \lambda\tau_s \cot\left(\frac{\hbar}{2k_{\mathrm{B}}T\tau_s}\right)\left[e^{-t/\tau_s} + \frac{t}{\tau_s} - 1\right] + \frac{4\lambda k_B T}{\hbar\tau_s}\sum_{n=0}^{\infty}\frac{\exp(-\nu_n t) + \nu_n t - 1}{\nu_n\left(\nu_n^2 - \frac{1}{\tau_s^2}\right)} \tag{13}$$

$$\nu_n = \frac{2\pi k_B T}{\hbar} n \tag{14}$$

$$g_I(t) = -\lambda\tau_s\left[e^{-\frac{t}{\tau_s}} - 1\right] \tag{15}$$

where λ is the reorganization energy, τ_s is the relaxation time, T is the temperature, k_B is the Boltzmann constant and $\hbar$ is the reduced Planck constant.

The impulsive fluorescence lineshape $f_{\text{imp}}(t, \omega_F)$ is obtained by computing $R_{\text{F}}(0, t, t_F)$, performing a Fourier transform along t_F, and taking the real part. Despite the somewhat lengthy equations, it has only 3 parameters: the transition energy $\Delta E = \hbar\omega_{eg}$, the reorganization energy λ and the relaxation time τ_s. This model thus relates the fluorescence peak shift to a microscopic model.

The next step is to account for population decay and finite experimental time resolution (temporal IRF). This is necessary as the IRF, peak shift and population decay all occur with 100 fs to 1 ps timescale. The population decay rate and IRF width are both obtained from the band integral of the FLUPS signal over the entire bandwidth, as detailed in the previous sections. They are included in the peak shift model as follows. First, the impulse response is multiplied by $K(t)$, and the resulting product is convolved with $F(t)$:

$$f_{\text{F}}(t, \omega) = F(t) * \left[K(t) \cdot f_{\text{imp}}(t, \omega)\right]. \quad (16)$$

The barycenter of $f_F(t, \omega)$ is then computed for each time, mirroring the data analysis performed for the experimental data. The resulting curve is fitted in a least-square sense by varying the lineshape parameters: ΔE, λ and τ_s. We use surrogate-accelerated curve-fitting to achieve convergence in a lower number of function evaluations. This fit produces the black curve on Fig. 3(b) of the main manuscript. The fit results for n-hexane are highly similar. Table S2 shows the best-fit parameters for both solvents.

A similar approach was tried with a damped harmonic oscillator, setting $C(\omega)$ to a Lorentzian spectral distribution in equation (11). However, the initial inertial regime does not fit the data. The best fit has double the residuals of the overdamped model, and unphysically high values of the reorganization energy. Therefore, we conclude the overdamped oscillator model best describes the peak shift and the corresponding microscopic dynamics.

Table S2. Best fit parameters for the overdamped oscillator model.

Solvent	$\boldsymbol{\Delta E}$ (eV)	$\boldsymbol{\lambda}$ (eV)	$\boldsymbol{\tau_s}$ (ps)
acetonitrile	2.909	0.3585	0.2341
n-hexane	2.934	0.3826	0.2248

S9. Population kinetics in TA

Our kinetic model follows the previous works on stiff-stilbene. It has the following elementary steps and rates:

$$Zs^* \rightarrow P, \qquad k_1 \quad (17)$$

$$P \rightarrow Zs, \qquad k_Z \quad (18)$$

$$P \rightarrow Em, \qquad k_E \quad (19)$$

We define $k_2 = k_{\text{Z}} + k_{\text{E}}$. In n-hexane, there seems to be an inverse reaction for the first step, but it is not discussed further here.

From conservation of mass, we have for all times:

$$[Zs] + [Zs^*] + [P] + [E\text{m}] = c \tag{20}$$

where c is the initial concentration of molecular motor.

As initial conditions, we assume only Zs^* is populated. The solution to this system is easily derived:

$$[Zs^*](t) = [Zs^*]_0 \exp(-k_1 t) \tag{21}$$

$$[P](t) = \frac{k_1 [Zs^*]_0}{k_1 - k_2} (\exp[-k_1 t] - \exp[-k_2 t]) \tag{22}$$

$$[E\text{m}](t) = \frac{k_E k_2 [Zs^*]_0}{k_1 - k_2} \left(\frac{\exp[-k_1 t]}{k_1} - \frac{\exp[-k_2 t]}{k_2} \right) + [E\text{m}]_\infty \tag{23}$$

and $[Zs](t)$ is given by equation (20) above. The equation for $[Zs^*]$ is a single exponential decay with rate constant k_1 and all other kinetics follow biexponential decay with rates k_1 and k_2.

The FLUPS band integral, discussed above, contains only the contribution from [Zs*], and the fit yields k_1 directly. The band integrals of the TA data are computed as:

$$I(t) = \frac{1}{\ln(\lambda_2/\lambda_1)} \int_{\lambda_1}^{\lambda_2} \frac{\Delta A(\lambda, t)}{\lambda} \mathrm{d}\lambda \tag{24}$$

The bounds, shown on Fig. 2B of the manuscript, were chosen as follows. The G band tracks the reduction in [Zs]: $I_G \propto c - [Zs](t)$. The P/Em band contains contributions from both the P intermediate and the Em band, weighted by their relative contributions to the band integral, ie: $I_{\mathrm{P}/E\mathrm{m}} = I_P[\mathrm{P}](t) + I_{E\mathrm{m}}[E\mathrm{m}](t)$. The final result is that the band integrals of TA can be fitted by biexponential models with two components: k_1 and k_2, whose intensities are given by the kinetic equations above, scaled by their unknown contribution to the band integrals. As such, we use biexponential models with arbitrary amplitudes and time constants $\tau_j = 1/k_j$.

The band integrals are fitted globally with the following kinetic model:

$$I_i(t) = F(t) * \left(\sum_j a_{ij} K_j(t) + y_i \right) \tag{25}$$

$$K_j(t) = \exp\left(-t/\tau_j\right) \tag{26}$$

Where i indexes the band integrals: 0 for G and 1 for P/Em, $F(t)$ is a gaussian IRF kernel of width σ_t and position t_0 as in eq. (4), a_{ij} is a matrix of amplitudes, K_j is an exponential decay component with time constant τ_j and y_i is the value of the band integral at long times.

We assume two population conversion times $\tau_i = 1/k_i$ as described above. A third component τ_0 is added to account for the shift of the SE out of the P/Em band integral window at very early times; this component is not used for the G band and $a_{00} = 0$. Furthermore, the G band has contributions only from Zs and Em, such that:

$$\left. \frac{dI_0}{dt} \right|_{t=0} \propto [\mathrm{P}](\mathrm{t} = 0) = 0, \tag{27}$$

as photoexcitation produces Zs^* only. This provides a constraint such that:

$$a_{01} = -a_{02}\frac{\tau_1}{\tau_2} \tag{28}$$

The value of τ_1=0.8303 ps is obtained from fitting the FLUPS band integral and t_0=0, as described previously. As such, the model has 9 free parameters. The fit produces the value τ_2=3.96(10) ps.

S10. Details of quantum-chemical calculations

Quantum-chemical calculations were carried out with the use of the Firefly v. 8.2 package[11] partly based on the GAMESS(US) source code[12]. We used the XMCQDPT2 quasi-degenerate multiconfiguration perturbation theory[13] on top of the CASSCF calculations with the Def2-TZVP triple-zeta basis set[14]. Intruder state avoidance (ISA) shift of the denominators with the ISA parameter of 0.02 a.u. was used throughout the calculations. Our tests demonstrated good stability of the results with respect to varying the ISA shift.

Optimization of the S_0 and the S_1 state were carried out with single-state CASSCF references, i.e. effectively in the MR-MP2 regime. In those optimizations, the active space consisted of 10 electrons in 10 p-orbitals, disregarding the low-lying lone pairs of the oxygen atoms and excluding the two lowermost p-orbitals with population of above 1.95 and the two uppermost ones with population of below 0.05. With explicit use of the C_2 symmetry, the ground state was optimized within the A irreducible representation and the excited state as the lowest state of the B irreducible representation. In terms of internal coordinates, the S_1 state demonstrates, in particular, elongation of the central ethylenic bond, shortening of the adjacent C(Et)-C(Ph) bonds, an increase in the central dihedral angle, and flattening of the dihedral angles between the central ethylenic bond and the phenyl rings. The integral effect of those changes on the molecular shape and volume is rather small. We note that both geometries are more open than either ground state or relaxed excited state geometries found in the parent stiff-stilbene, due to the increased steric interaction.

Single-point calculations of the relative energy of the S_0 and the S_1 states were carried out with the complete p-orbital active space of 14 electrons and with the RI approximation to speed up the computations. Since we intended to cover both states within one common computation, no symmetry was used. The calculations involved 6-state averaged CASSCF reference needed to properly cover the strongly absorbing states at the ground state geometry where considerable reordering of the excited states is observed at the CASSCF level.

It was found that the oscillator strength in the lowest electronic transitions is mostly associated with the single-electron HOMO-LUMO excitation. At the geometry of the ground state, XMCQDPT2 predicts the S_1 state at 4.03 eV to be dark, and the HOMO-LUMO excitation to contribute to S_2 at 4.04 eV and S_3 at 4.40 eV with a ratio of oscillator strengths of ca. 0.27:0.65. One should note, however, that the respective computational results can be very sensitive to the exact details of the computational model when the excitation of interest strongly interacts with other transitions and thus mixes with them. At the optimized geometry of the S_1 state, it is dominated by the HOMO-LUMO excitation, the vertical emission energy being 2.66 eV, with oscillator strength of 0.25. The energy of the 0-0 transition is predicted to be 3.59 eV, and the difference in

the energy of S_0 between the two geometries is about 0.93 eV. These results are compared to experimental spectra in Figure S10.

S11. Derivation of the effective equation of motion

We derive the effective equation of motion for a single degree of freedom, subject to fluctuation and dissipation arising from coupling to the environment. We start with the Langevin equation of motion for a single degree of freedom[15]:

$$m\ddot{q} = -\frac{\partial U}{\partial q} - \gamma\dot{q} + \sqrt{2k_B T\gamma}\,\eta(t) \tag{29}$$

where m is the effective mass of mode q, U is the conservative potential, γ is the friction coefficient, k_B is the Boltzmann constant, T is the temperature and $\eta(t)$ is a random number with gaussian distribution, mean of 0 (ie: $\langle\eta(t)\rangle = 0$), unit variance (ie: $\mathrm{Var}[\eta(t)] = 1$) and where consecutive values are uncorrelated (ie: $\langle\eta(t)\eta(0)\rangle \propto \delta(t)$).

For our model, we assume $U = k(q - q_0)^2/2$. Reorganizing the equation, we get:

$$0 = -m\ddot{q} - k(q - q_0) - \gamma\dot{q} + \sqrt{2k_B T\gamma}\,\eta(t) \tag{30}$$

Neglecting inertia, ie: $m\ddot{q} \approx 0$, and reorganizing yields:

$$\dot{q} = -\frac{k}{\gamma}(q - q_0) + \sqrt{\frac{2k_B T}{\gamma}}\,\eta(t) \tag{31}$$

In the manuscript, we assume the reaction coordinate maps to the dihedral angle (ie: $q{\sim}\varphi$).

This equation describes the evolution of a stochastic degree of freedom in the overdamped limit. It is also known as the Ornstein-Uhlenbeck process. It is the process that gives rise to the lineshape function given by eqs. (12)-(15). This process has been thoroughly studied[7,15,16].

For example, it lets us estimate the evolution of the width of the distribution of angles during relaxation. In our harmonic oscillator approach, the spring constant is related to the energy difference and the difference of dihedral angles as $k = 2\Delta E/\Delta\varphi^2$. The standard deviation of a harmonic oscillator at thermal equilibrium is $\sigma_{\mathrm{eq}} = \sqrt{k_\mathrm{B}T/k}$. Our QCC calculations indicate $\Delta\varphi$=27.7°, $\Delta E(S_0)$=0.93 eV and $\Delta E(S_1)$=0.45 eV. This yields $\sigma_{\mathrm{eq}}(S_0)$=3.2° and $\sigma_{\mathrm{eq}}(S_1)$=4.7°. After photoexcitation, the average angle will evolve from 3.0° to 30.7°, and the widths of the distribution evolve from 3.2° to 4.7°. For the Ornstein-Uhlenbeck process, the average and variance evolve as[16]:

$$\langle\varphi(t)\rangle = \varphi_0 + \left(\varphi_{\mathrm{eq}} - \varphi_0\right)\left(1 - e^{-t/\tau}\right) \tag{32}$$

$$\sigma^2(t) = \sigma_0^2 + \left(\sigma_{\mathrm{eq}}^2 - \sigma_0^2\right)\left(1 - e^{-2t/\tau}\right) \tag{33}$$

The curves on Figure 4D of the manuscript were obtained from these equations.

References

(1) Kathan, M.; Crespi, S.; Troncossi, A.; Stindt, C. N.; Toyoda, R.; Feringa, B. L. The Influence of Strain on the Rotation of an Artificial Molecular Motor. *Angew Chem Int Ed* **2022**, *61* (34), e202205801. https://doi.org/10.1002/anie.202205801.

(2) van Leeuwen, T.; Gan, J.; Kistemaker, J. C. M.; Pizzolato, S. F.; Chang, M.; Feringa, B. L. Enantiopure Functional Molecular Motors Obtained by a Switchable Chiral-Resolution Process. *Chemistry A European J* **2016**, *22* (21), 7054–7058. https://doi.org/10.1002/chem.201600628.

(3) Van Leeuwen, T.; Neubauer, T.; Feringa, B. Regioselective Synthesis of Indanones. *Synlett* **2014**, *25* (12), 1717–1720. https://doi.org/10.1055/s-0033-1339156.

(4) Dobryakov, A. L.; Kovalenko, S. A.; Weigel, A.; Pérez-Lustres, J. L.; Lange, J.; Müller, A.; Ernsting, N. P. Femtosecond Pump/Supercontinuum-Probe Spectroscopy: Optimized Setup and Signal Analysis for Single-Shot Spectral Referencing. *Review of Scientific Instruments* **2010**, *81* (11), 113106. https://doi.org/10.1063/1.3492897.

(5) Kapitzke, M.; Palato, S.; Raj, A.; Kumpulainen, T.; Stähler, J. Enhancing Spectral Coverage, Efficiency, and Photometric Accuracy in Ultrafast Fluorescence Upconversion Spectroscopy. *ChemPhotoChem* **2025**, e202400398. https://doi.org/10.1002/cptc.202400398.

(6) Zhang, X.-X.; Würth, C.; Zhao, L.; Resch-Genger, U.; Ernsting, N. P.; Sajadi, M. Femtosecond Broadband Fluorescence Upconversion Spectroscopy: Improved Setup and Photometric Correction. *Review of Scientific Instruments* **2011**, *82* (6), 063108. https://doi.org/10.1063/1.3597674.

(7) Mukamel, S. *Principles of Nonlinear Optical Spectroscopy*; Oxford University Press: New York, NY, 1995; Vol. 6.

(8) Li, B.; Johnson, A. E.; Mukamel, S.; Myers, A. B. The Brownian Oscillator Model for Solvation Effects in Spontaneous Light Emission and Their Relationship to Electron Transfer. *Journal of the American Chemical Society* **1994**, *116* (24), 11039–11047. https://doi.org/10.1021/ja00103a020.

(9) Palato, S. Multimode Brownian Oscillator. *https://github.com/spalato/Mbo.jl*, 2018. https://github.com/spalato/Mbo.jl.

(10) Hamm, P.; Zanni, M. *Concepts and Methods of 2D Infrared Spectroscopy*; Cambridge University Press: Cambridge, 2011; Vol. 9781107000.

(11) Granovsky, A. A. *Firefly version 8*. http://classic.chem.msu.su/gran/firefly/index.html.

(12) Schmidt, M. W.; Baldridge, K. K.; Boatz, J. A.; Elbert, S. T.; Gordon, M. S.; Jensen, J. H.; Koseki, S.; Matsunaga, N.; Nguyen, K. A.; Su, S.; Windus, T. L.; Dupuis, M.; Montgomery, J. A. General Atomic and Molecular Electronic Structure System. *J. Comput. Chem.* **1993**, *14* (11), 1347–1363.

(13) Granovsky, A. A. Extended Multi-Configuration Quasi-Degenerate Perturbation Theory: The New Approach to Multi-State Multi-Reference Perturbation Theory. *The Journal of Chemical Physics* **2011**, *134* (21), 214113. https://doi.org/10.1063/1.3596699.

(14) Weigend, F.; Ahlrichs, R. Balanced Basis Sets of Split Valence, Triple Zeta Valence and Quadruple Zeta Valence Quality for H to Rn: Design and Assessment of Accuracy. *Phys. Chem. Chem. Phys.* **2005**, *7* (18), 3297. https://doi.org/10.1039/b508541a.

(15) Chandler, D. *Introduction to Modern Statistical Mechanics*; Oxford University Press: New York-Oxford, 1987.
(16) Kampen, N. G. van. *Stochastic Processes in Physics and Chemistry*, 3. ed.; North-Holland personal library; Elsevier: Amsterdam, 2007.

Supplementary figures

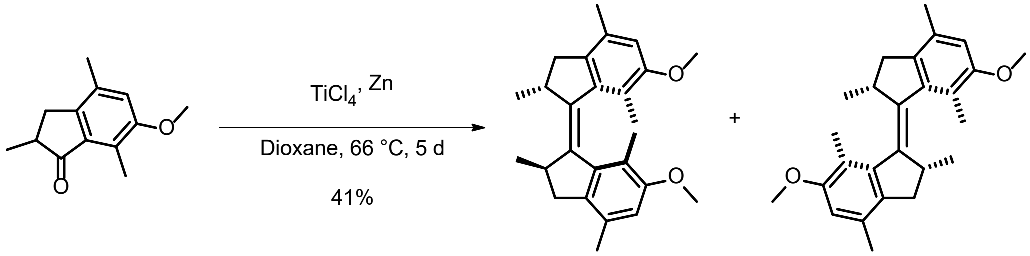

Fig. S1. McMurry coupling towards motor *Z*s and *E*s. Only (*R*,*R*)-enantiomer is shown for clarity, although the motor was obtained as a racemic mixture which also contains the (*S*,*S*)-enantiomer. The (*S*,*R*)-meso compound is not formed during the McMurry coupling. The starting indanone was obtained via a literature procedure.

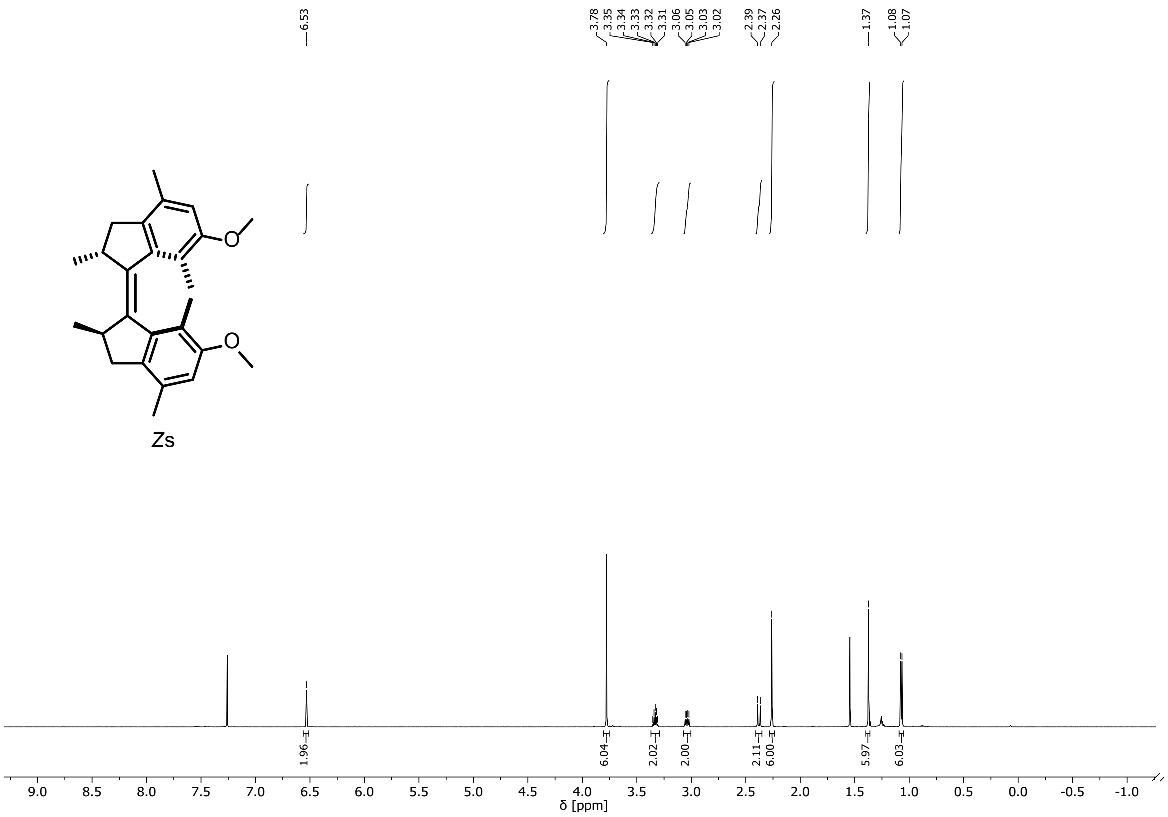

Fig. S2. **^{1}H NMR** Spectrum (600 MHz, 25 °C) of *Z*s in $CDCl_3$.

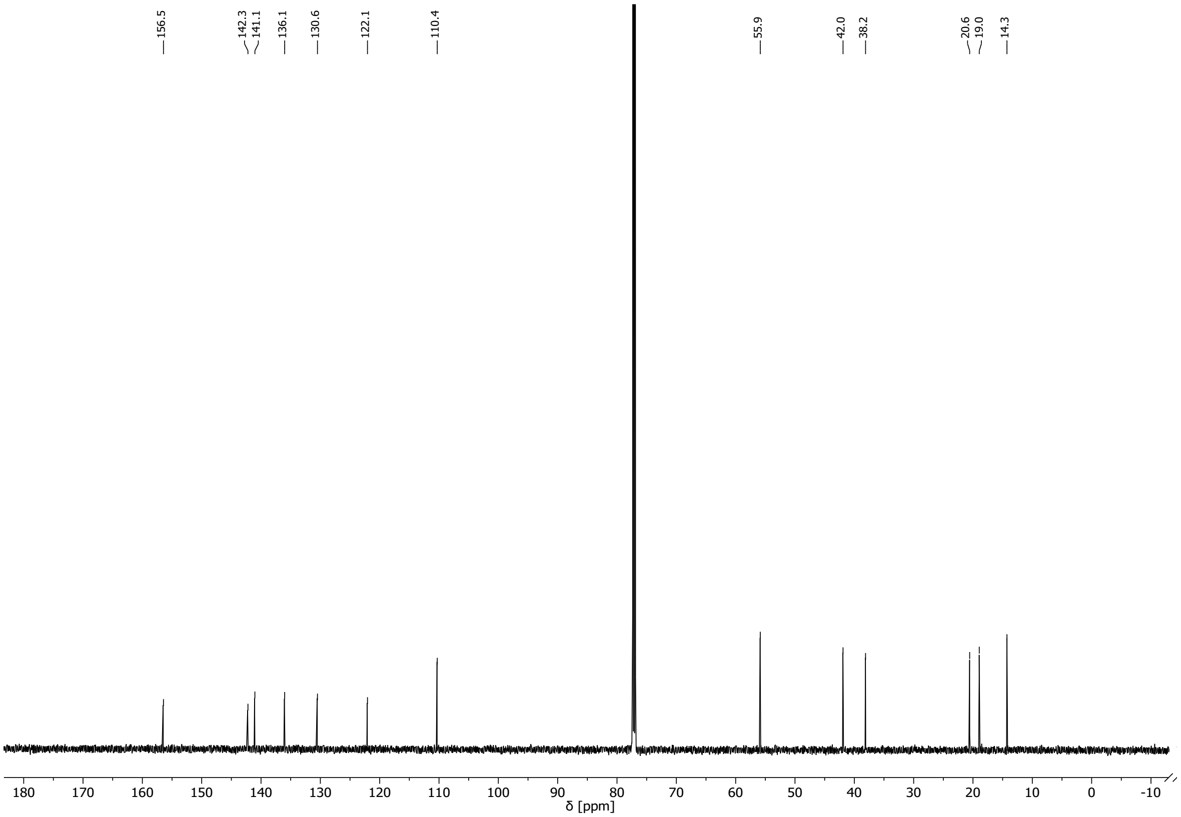

Fig. S3. $^{13}C\{^{1}H\}$ **NMR** Spectrum (151 MHz, 20 °C) of *Z*s in $CDCl_3$.

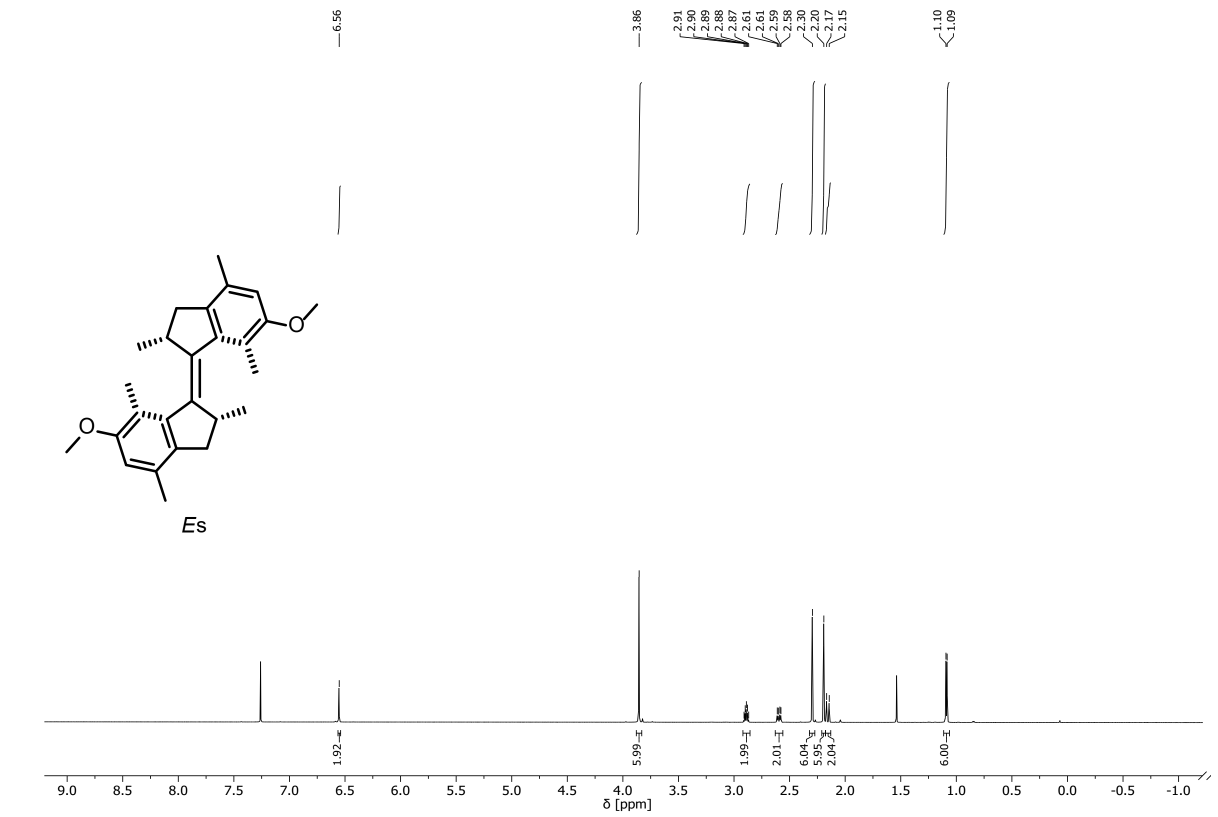

Fig. S4. [1]**H NMR** Spectrum (600 MHz, 25 °C) of *E*s in $CDCl_3$.

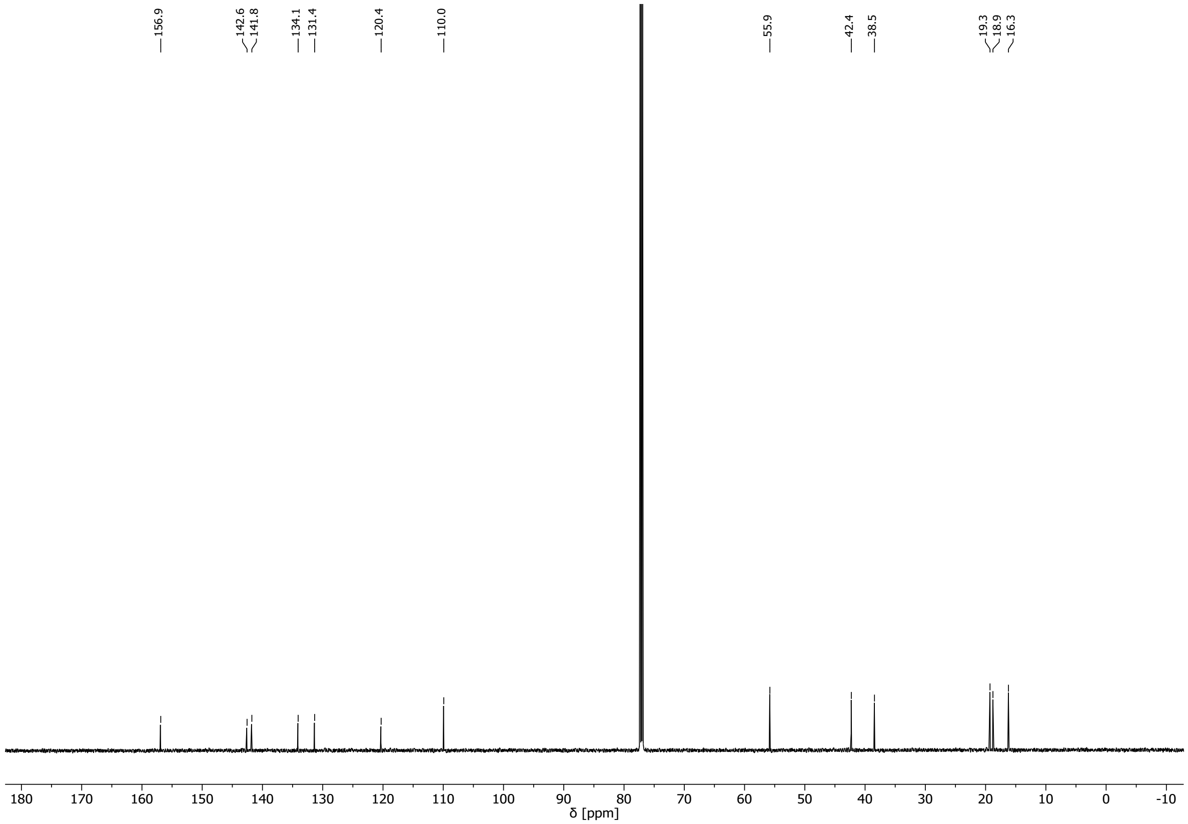

Fig. S5. ^{13}**C{**^{1}**H} NMR** Spectrum (151 MHz, 25 °C) of *E*s in $CDCl_3$.

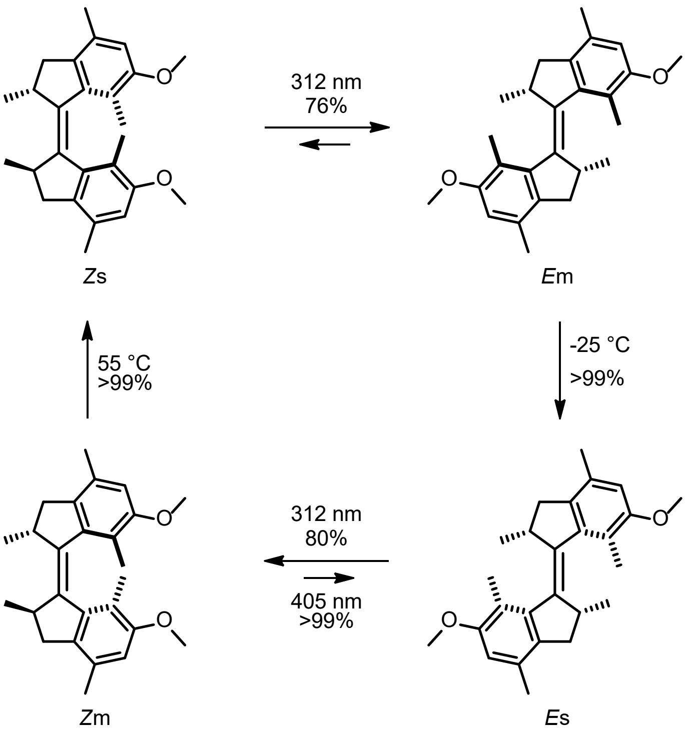

Fig. S6. Rotational cycle of motor isomer *Z*s. Irradiation of motor *Z*s with 312 nm UV light induces a *Z*/*E* isomerization forming motor isomer *E*m. Subsequent THI results in the formation of motor isomer *E*s. Another irradiation with 312 nm UV light induces an *E*/*Z* isomerization forming isomer *Z*m that subsequently undergoes THI forming the initial *Z*s isomer. Only (*R*,*R*)-enantiomer is shown for clarity. Unidirectionality of this motor has been indicated in literature (*14*).

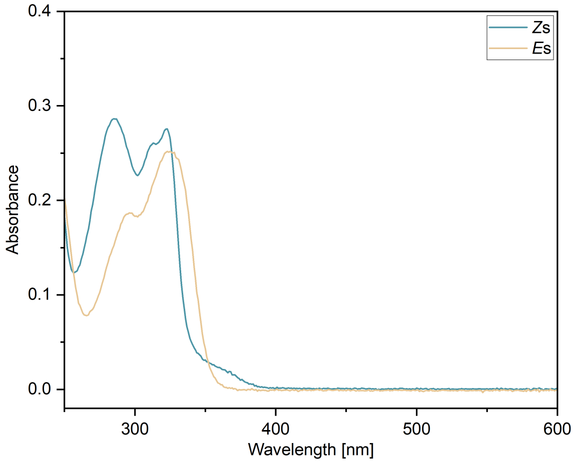

Fig. S7. Overlapping UV/vis spectra of motor *Z*s and *E*s in MeCN/Water/IPA (9:2.5:1 + 0.1% formic acid) at 20 °C, c = ~20 µM. Teal = motor *Z*s, Orange = motor *E*s. Isosbestic point at 326 nm.

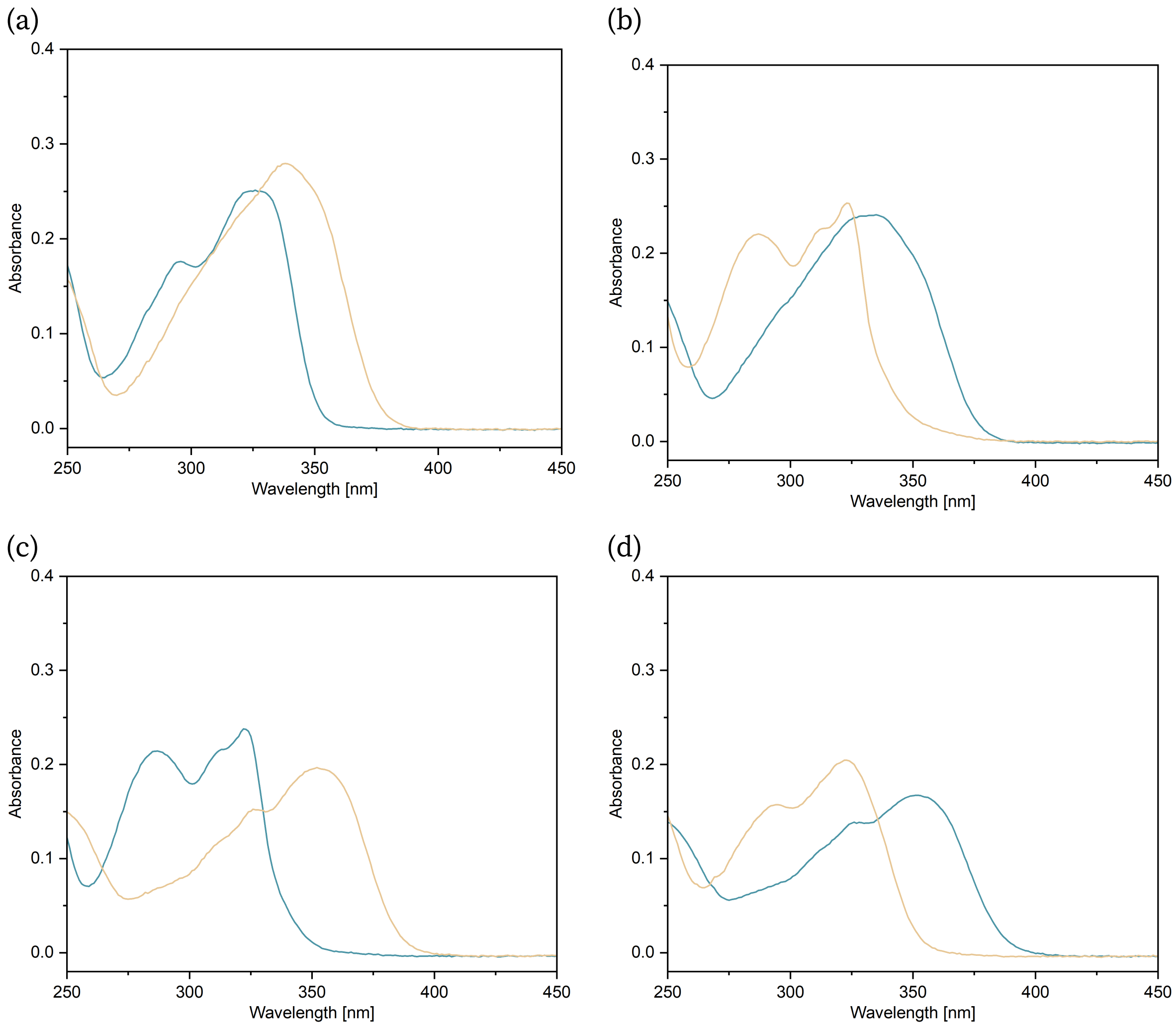

Fig. S8. Rotation cycle of motor *Z*s followed by UV/vis spectroscopy in MeCN, c = ~10 µM. From teal to orange. (a) photochemical *Z*/*E* isomerization of *Z*s to *E*m, 308 nm, –40 °C, 76% PSS. (b) THI of motor *E*m to *E*s at –25 °C. (c) photochemical *Z*/*E* isomerization of *E*s to *Z*m, 308 nm, 0 °C, 80% PSS. (d) THI of motor *Z*m to *Z*s at 55 °C. PSS ratios were determined by UPLC-HRMS.

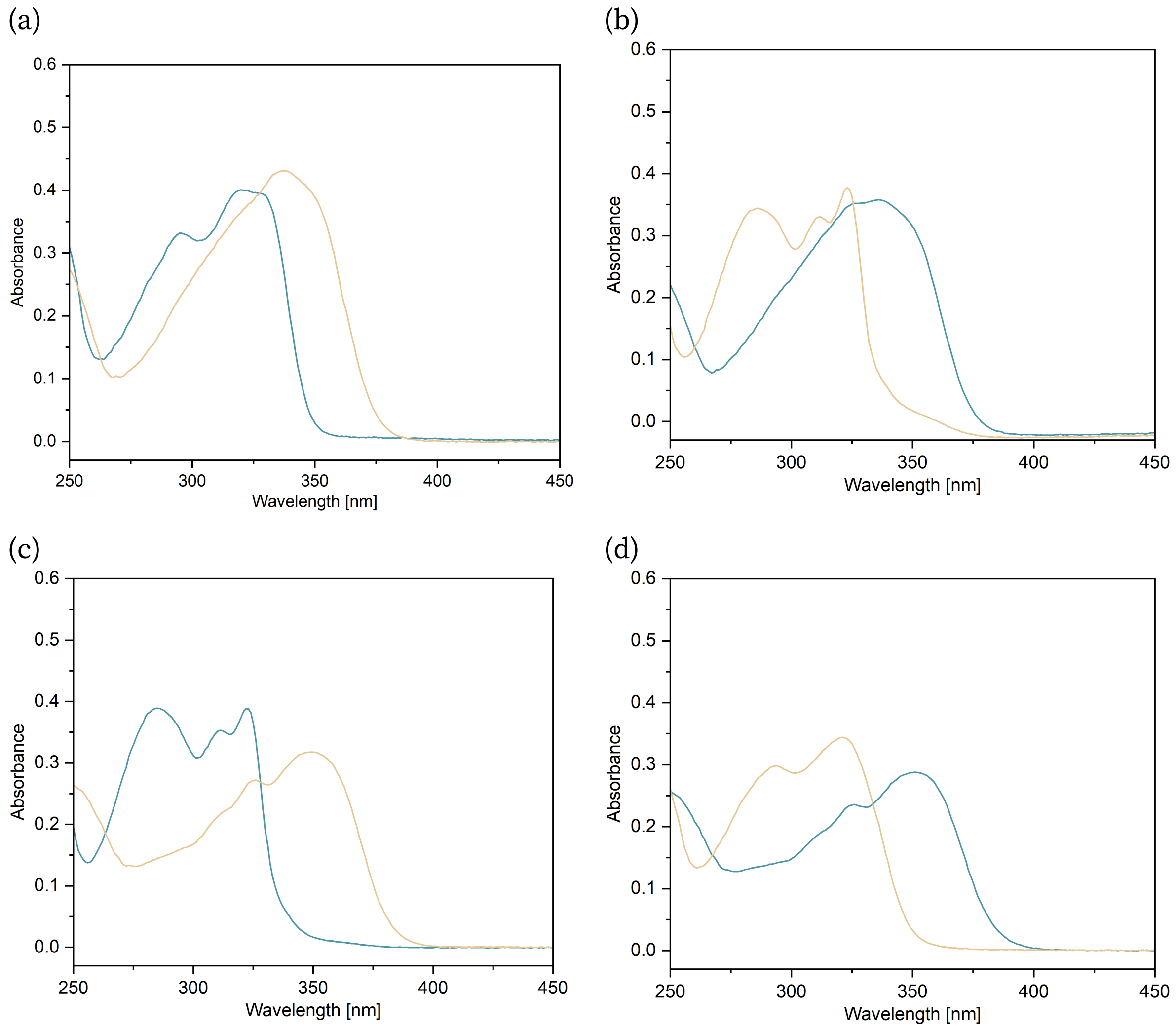

Fig. S9. Rotation cycle of motor *Z*s followed by UV/vis spectroscopy in n-hexane, c = ~10 µM. From teal to orange. (a) photochemical *Z*/*E* isomerization of motor *Z*s to *E*m, 308 nm, –40 °C, 77% PSS. (b) THI of motor *E*m to *E*s at –25 °C. (c) photochemical *E*/*Z* isomerization of motor *E*s to *Z*m, 308 nm, 0 °C, 81% PSS. (d) THI of motor *Z*m to *Z*s at 55 °C. PSS ratios were determined by UPLC-HRMS.

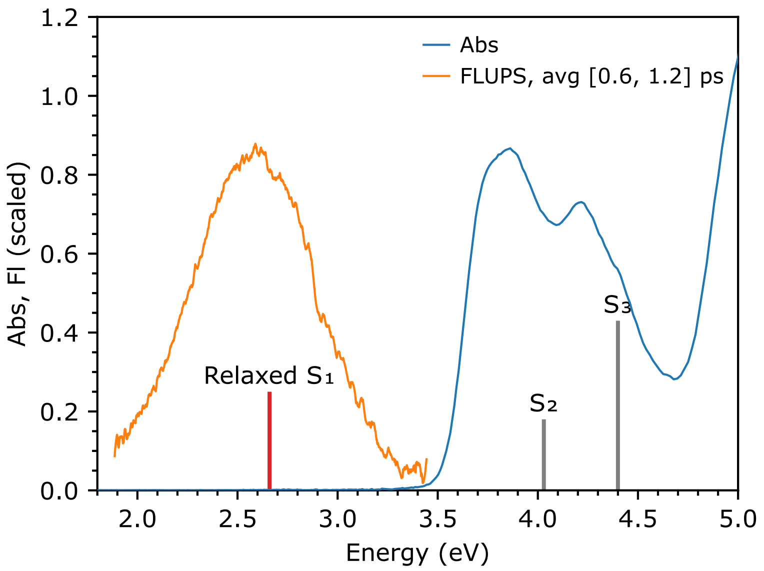

Fig. S10. Comparison of absorption spectrum (blue), relaxed fluorescence (orange) and calculated transition energies (grey, red bars).
